# Developing a Mood and Menstrual Tracking App for People With Premenstrual Dysphoric Disorder: User-Centered Design Study

**DOI:** 10.2196/59333

**Published:** 2024-12-24

**Authors:** Chloe Apsey, Arianna Di Florio, Katarzyna Stawarz

**Affiliations:** 1 Division of Psychological Medicine and Clinical Neurosciences, School of Medicine, Cardiff University, Cardiff, United Kingdom; 2 National Centre for Mental Health, Cardiff University, Cardiff, United Kingdom; 3 School of Computer Science, Cardiff University, Cardiff, United Kingdom

**Keywords:** premenstrual dysphoric disorder, menstrual tracking, mood tracking, mobile health, mHealth, user-centered design, menstrual, tracking app, hormonal fluctuations, mood monitoring, menstruation

## Abstract

**Background:**

People with premenstrual dysphoric disorder (PMDD) experience a range of symptoms that increase and decline as a result of the natural hormonal fluctuations of the menstrual cycle. For the diagnosis of PMDD, symptom severity needs to be recorded daily for at least two symptomatic cycles. In recent years, the rise in interest in Femtech (tools and technology developed to address women’s health issues) has resulted in a large quantity of “period-tracking apps” being developed and downloaded. However, there is not currently a menstrual and mood tracking app that has the full capabilities to accurately capture the symptoms of PMDD to aid with diagnosis.

**Objective:**

This study aimed to collect feedback and insights from potential users (ie, people with lived experience of PMDD or severe premenstrual syndrome) to inform the development of a prototype app that could support prospective mood monitoring of PMDD symptoms for research, and to support diagnosis.

**Methods:**

We conducted two user-centered design studies. Study 1 consisted of 4 interviews with individual participants who had taken part in our previous web-based mood tracking study for PMDD. During the interviews, participants were encouraged to identify the strengths and weaknesses of the existing web-based mood tracking system. Study 2 consisted of 2 workshops with a total of 8 participants, in which participants were asked to discuss the needs and desirable features they would like in a PMDD-specific tracking app. Interviews and workshops were recorded, and the transcripts were analyzed inductively following a thematic approach.

**Results:**

A total of four themes were identified from the interviews and workshops with potential users: (1) ease of use as a key consideration for users with PMDD; (2) avoiding a reductionist approach for a broad range of symptoms; (3) recognizing the importance of correct language; and (4) integrating features for the users’ benefits. These suggestions align with the current understanding of the implications of PMDD symptoms on daily activities and with findings from previous research on encouraging long-term engagement with apps.

**Conclusions:**

To meet the needs of potential users with PMDD or suspected PMDD, there needs to be a special consideration to how their symptoms impact the way they might interact with the app. In order for users to want to interact with the app daily, particularly during the days where they may not have symptoms to track, the app needs to be simple yet engaging. In addition, if the app provides insights and feedback that can benefit the well-being of the users, it is suggested that this could ensure prolonged use.

## Introduction

Premenstrual dysphoric disorder (PMDD) affects 1%‐5.5% of people with periods [1]. It is defined as the sudden onset of severe changes in emotional, cognitive, and behavioral functioning during the late luteal phase of the menstrual cycle (ie, the 7‐10 d before the onset of menstrual bleeding). Symptoms subside within a few days of the onset of menses [2] and are significantly distressing, with 30% of people with PMDD attempting to commit suicide in their lifetime according to a survey [3].

The American Psychiatric Association’s *Diagnostic and Statistical Manual of Mental Disorders, Fifth Edition* (*DSM-5* [2]) and World Health Organization’s *International Classification of Diseases, Eleventh Revision* (*ICD-11* [4]) diagnostic criteria for PMDD specify that symptoms need to be confirmed prospectively by daily ratings recorded for at least 2 symptomatic cycles. The Daily Record of Severity of Problems (DRSP [5]) is a validated rating scale for the prospective mood and cycle tracking. Together with the Carolina Premenstrual Assessment Scoring System (C-PASS [6]), it facilitates a standardized approach to the diagnosis of PMDD, based on internationally recognized diagnostic criteria. While retrospective reports of PMDD symptoms yield high rates of false positives [5], there is often a complete failure to consider prospective daily ratings. Challenges and limitations to reliable prospective mood ratings include high dropout rates [7], the high costs, and the uncertainties around the prospective nature of self-report (ie, patients can go back and change scores) [8].

Mobile phone apps have become a common feature to many in modern life. For research, diagnosis and interventions, apps can be extremely effective due to the potential to be flexible, tailored, customizable, wide-reaching, cost effective; and commonly accepted as a means of health promotion [9]. Furthermore, apps can incorporate the most effective components of behavior change when sufficient planning has taken place to ensure that it user-friendly, engaging, and flexible [10]. Menstrual tracking apps have high engagement [11] and new research is focusing on their design, intended use and potential unintended impact [12,13].

The majority of people who use tracker apps download them with the purpose of tracking their bleeding to receive predictions of the future cycle and to predict their fertility window [14]. However, evidence shows these apps rarely consider the variability in cycle length meaning predictions are often inaccurate [15], which has shown to have implications on the user’s well-being, making them feel “abnormal” [16]. In addition, as of March 2024, only a few dedicated PMDD apps exist (eg “Me vs. PMDD”), and there is scant research on PMDD apps at the moment.

While hundreds of regular menstrual tracking apps are available [17,18], it is unclear whether they could be effectively used to support PMDD, especially since most of them do not provide standardized questionnaires necessary for reliable diagnosis such as the DRSP [5]. Given the limitation of the current apps for menstrual tracking and the clinical importance of prospective rating for a valid and reliable diagnosis of PMDD, we sought to coproduce an app with people with lived experience. In order to ensure this app has high levels of usability, we aimed to modify an existing survey based on initial feedback from potential end users, which is a widely recommended and previously successful approach [19]. It is important that we overcome the existing concerns of menstrual tracking apps by being open to individual differences in menstrual cycle length while also ensuring a high standard of data security for our users.

This paper reports the results of interviews and workshops with people with lived experience to (1) explore user pain points and issues with usability and engagement and (2) identify user needs and desirable features that would support engagement with a diagnostic tool that requires continued use for at least 2 months.

## Methods

### Design

We conducted 2 user-centered studies with people with lived experience of severe premenstrual syndrome (PMS) or PMDD. User-centered design methods actively encourage participants to make suggestions with regard to the potential design and functionality of the system [20], which is necessary when designing systems for participant groups with varied experience and symptoms. In total, 2 series of discussions (one set of interviews and one set of workshops) were organized. In the first (Study 1), the focus of the discussion was barriers to engagement and factors responsible for the high drop-out rates using the current monitoring tool available online through our website [21]. In the second (Study 2), we gathered ideas and suggestions from stakeholders to cocreate an ideal app during online workshops.

### Participants and Recruitment

For both studies, participants were recruited via the mailing list from our ongoing research program on the biological basis of PMDD, “Premenstrual Dysphoric Disorder; Indicators Causes and Triggers (PREDDICT)” [21]. For people who believe they have PMDD, but who have never received a formal diagnosis, PREDDICT offers the opportunity to complete either a web-based or a paper version of the DRSP [5], modified to include a question on suicidality.

Participants to both studies were required to be aged 18 years or older, to understand either spoken written English or Welsh or both, and be able to provide valid informed consent. Written informed consent was obtained from all participants, and participants were encouraged to keep their name and personal details private during interviews or workshops.

The participants were invited to take part in Study 1 if they had taken part into PREDDICT web-based mood monitoring with the modified DRSP [22] and had completed a minimum of 4 days. A mix of people who had successfully completed the diary for the required 2 months, in addition to those who dropped out, were invited to participate. The participants were invited to take part in Study 2 if they had lived experience of severe PMS or PMDD. A total of 515 people were on our mailing list for the study invite, including those invited to join Study 1. The participants were allocated to Study 2 based on their availability to attend online workshops on the given day.

Out of the 18 participants who were available on the given dates, 12 had been diagnosed with PMDD by a health care professional using a mood-menstrual diary and 6 self-reported experience severe PMS or PMDD but have never received a formal diagnosis.

For Study 1, 4 participants attended the interviews. For Study 2, a total of 8 participants attended the 2 workshops. For workshop 1, only 1 person attended (5 people were due to attend but dropped out on the day due to feeling unwell). As for workshop 2, 7 participants attended. Of the 18 participants, 1 participant (5.5%) was aged between 18 and 25 years, 6 were aged between 26 and 33 years, 6 between 34 and 41 years, 4 between 42 and 50 years, and 1 was 50+.

### Materials for Both Studies

All participants were provided with a participant information sheet (Multimedia Appendix 1) prior to signing the written consent form (Multimedia Appendix 2). The participants were also all asked to fill out a demographic questionnaire (Multimedia Appendix 3) on Microsoft Forms that asked for age range, gender identity, ethnic origin, if they have received a PMDD diagnosis, and if they have completed a PMDD mood diary in the past. All study materials are available in the multimedia appendices.

### Procedures

#### Study 1: Interviews

The one-to-one interviews were conducted via Zoom and audio recorded (with participants’ consent given beforehand). Interviews lasted between 30 and 45 minutes and were conducted by CA. The interviewer followed an open interview guide (Multimedia Appendix 4) with questions on the web-based mood tracking system followed by questions on the participant’s experience with other menstrual and mood tracking apps, if any used, and what features they would ideally like to see in them.

For their participation, each participant received a Love2Shop voucher of £15 (US $19.30).

#### Study 2: Co-Design Workshops

Workshops were conducted online and facilitated by CA and KS. An online whiteboard, Miro, was used to visually map the discussions [23], allowing participants to add “Sticky Notes” containing text to the board during discussions.

The workshops were preplanned (Multimedia Appendix 5) and consisted of 3 activities that aimed to encourage discussions about (1) what apps people currently used, (2) what they did or did not like about them and the additional features they would add to make the app more useful, and (3) identifying 5 features that the ideal app should have.

For the second workshop, 7 people were present, and breakout groups of 2 or 3 people were created to allow everybody to contribute. The participants were then brought back to the main group to discuss their ideas and add their suggestions to sticky notes on a Miro board [23].

The workshop group discussion was audio recorded (with prior consent from the participants). At the end of each workshop, each attendee received a £20 (US $25.73) Love2Shop voucher.

### Ethical Considerations

This research project has been reviewed and given a favorable opinion by the COMSC Research Ethics Committee, Cardiff University (project COMSC/Ethics/2023/042). The participants were provided with a participant information sheet prior to the study and invited to ask any questions they may have. If they were happy to take part, they were provided with a consent form to sign and reminded they have the ability to withdraw from the study at any stage.

### Analysis

The recordings from the interviews (Study 1) were transcribed, and the transcripts were analyzed inductively using Braun and Clarke’s reflexive thematic analysis approach [24]. One researcher (CA) completed the initial coding process to establish the relevant labels. More specifically, the steps involved the researchers pulling significant quotes from participants and adding them as “sticky notes” onto a new Miro board. This process was replicated with the transcripts from the workshops (Study 2) recordings, with the “sticky notes” being added to the same Miro board. Furthermore, the “sticky notes” recorded during the 3 activities completed during the workshops were also added to this new Miro board and randomized for affinity mapping [25].

Researchers (CA and KS) then went through each of the sticky notes and grouped them by affinity, based on content. These small groups were then discussed by the researchers to decide overarching themes that have arisen from these topics. On the Miro board, the themes were organized into boards, and all the individual sticky notes were organized into them; this allowed the researcher to do any revision based on discussions and to see if any notes did not conform to the suggested themes or if themes could be grouped any further. The analysis resulted in 4 themes.

## Results

We identified 4 themes for consideration when designing an app for the tracking of PMDD symptoms. These themes are (1) ease of use as a key consideration for users with PMDD, (2) avoiding a reductionist approach for a broad range of symptoms, (3) recognizing the importance of correct language, and (4) integrating features for the users’ benefits. They are described below with illustrative quotes.

### Theme 1: Ease of Use as a Key Consideration for Users With PMDD

During both the interviews and workshops, the participants alluded to the importance of ease of use to ensure prolonged engagement.

As implied by the diagnostic criteria of PMDD, during the symptomatic luteal phase, the individuals may experience increased irritability, depressed mood, or difficulty concentrating. If the app interface is not developed, considering the implications of the symptoms of PMDD, uploading symptoms ratings can feel like an additional burden. Completing surveys can be very time consuming and often some of the statements may not be relevant so can feel like time is wasted.

*it’s absolutely the bane of my life even on that 10-minute break filling out what my period has been like these last few days is like pulling teeth, I haven’t got time, patience, energy*

[Workshop (W) 2, Participant (P) 3; about adding in symptoms ratings on the existing app Clue]

Moreover, during the nonsymptomatic stages of the menstrual cycle, the participants suggested that they often do not think about nor want to think about the negative symptoms they experience during the luteal phase. Therefore, they do not interact with mood or menstrual tracking apps during this time.

*Exactly and you don’t even remember like sometimes I’m like god who was that because it’s just gone and once it’s gone, I’m like really relishing my good days so I don’t want to think about it, I don’t remember it*

[W2, P1 experiencing PMDD]

Across interviews and workshops, several attendees referenced their experience of having a comorbid diagnosis of attention-deficit/hyperactivity disorder (ADHD) or autism spectrum disorder. Recent research has implied an association between ADHD and PMDD [26]. Based on this, attendees highlighted that for neurodivergent people, an app needs to be visually stimulating and offer a range of accessibility features.

*To personalize it I think would really help because I know I just don’t have the attention span sometimes and I find, I don’t know if you guys are the same who have got the ADHD diagnosis but when I’m due on it is through the roof like the ADHD symptoms they are so much worse so like the time when you probably need to be recording things is the exact time it’s the last thing you want to do*

[W2, P1: having an ADHD diagnosis and PMDD]

### Theme 2: Avoiding a Reductionist Approach for a Broad Range of Symptoms

As mentioned before, there is a natural variability between people and even for individuals on a month-by-month basis. Naturally, this variation is also applicable for how and when PMDD symptoms may present. Therefore, when existing menstrual-tracking apps are not flexible with menstrual cycle length, it can lead to unnecessary distress and mistakes surrounding diagnosis.

*[W2, P7] and I were just saying that for both of us we didn’t have regular cycles so any apps that we tried to use were very much geared to people with regular cycles and in [P]’s case she was saying she even went a few months without a period whereas the apps would be telling you your period is due or your period is overdue*

[W2, P6; experience of irregular cycles]

An additional issue with menstrual and mood tracking apps that participants highlighted was the contextual nature of symptoms and the fact that their mood could change significantly throughout the day. As such, if they were only able to upload symptom rating once per day with no option to backtrack, the information could be biased by one’s mood at that very moment. Therefore, the option to input multiple entries for one day or the ability to change their entry would be preferred for better accuracy.

*Using my recollection to say how I felt through the day, was always tainted by how I was feeling at the time, because I think I was trying to be quite objective and be like, okay because it changes dramatically from one moment to the next*

[Interview (I) 4, rating symptoms]

The participants from both interviews and workshops also mentioned how the existing mood or menstrual tracking apps did not have a very comprehensive or PMDD-applicable tracking options when it came to mood. There often was a focus on either the physical or psychological symptoms but rarely both, meaning they could not accurately capture their changes in symptoms against their menstrual cycle stage. Therefore, there is a need for being able to track symptom severity and select which symptoms are relevant and users want to continue tracking.

*It’s mood and menstrual by the sounds of it because normally a lot of them tend to do one, or like I use Clue and they ask some little questions about like the mood and track it, but they’re quite like, you know, do you feel happy today or sad and that’s kind of it*

[I4, symptom tracking in existing apps]

### Theme 3: Recognizing the Importance of Correct Language

Workshop attendees spoke on the importance of scientific terminology and the negative implications of period “forecasting” when done incorrectly. Some participants reported that during their previous experiences using menstrual tracking apps, they found it triggering when an app informed them that they “should” be ovulating, but they were experiencing an irregular cycle. This evidenced how there needs to be an additional consideration to the way language is used in the context of a PMDD-specific menstrual or mood tracking app.

P3: *So, it would be nice to have that option to see it if you should be ovulating or if you aren’t ovulating because it isn’t, I’m not saying you don’t work but no one’s normal.*

P7: *It’s not black and white and if you’re feeling vulnerable it does make you think like there’s something wrong with you, you know more so.*

[W2, P3 and P7]

Furthermore, in both interviews and workshops, individuals mentioned how the language used in media to discuss the menstrual cycle can often be infantilizing and the use of euphemism can imply that the topic itself is a taboo. In order to prevent this, accurate and clear terminology needs to be used to support better conversations with medical professionals in the future and this should also be reflected in menstrual tracking apps.

*But I think it’s like being treated like an adult and it’s using the proper words not like making it all childish and things like that. Using the correct terminology. You can end up being quite childish about periods where people get it’s a mother’s curse or whatever. I’m exaggerating but let’s use proper terminology because they’re the terms that you want to go to the GP with and things like that.*

[W1, P1]

Moreover, language can be subjective and using scientific terminology (which as aforementioned is typically avoided when media talk about the menstrual cycle) can be confusing. For users to correctly interpret what the question is asking; images or additional descriptions need to be included to give better clarification.

*Oh, understanding what spotting meant, so I think I might have actually filled this out a little bit wrong at the beginning because what I understood as spotting was not actually, I think, the actual, what it means [laughs]. So having some clarification around the terminology would be good as well.*

[I4]

### Theme 4: Integrating Features for the Users’ Benefits

During all interviews and workshops, the participants emphasized that the ongoing use of apps are largely reliant on what benefits they gain from using it. In the context of menstrual or mood tracking apps, the reported main goal for using the app is to gain insights on the interaction between their cycle and the mood symptoms. Therefore, a visually interesting and intuitive graph or cycle summary that can be easily shared with partners, family, friends, or doctors, was highly requested.

*Some kind of data visualization way my mood and my physical symptoms and how they seem to correlate.*

[I3]

As the apps often ask about the presence of negative feelings that can be seen as intrusive and potentially triggering, the participants suggested integrating tools that may be uplifting or therapeutic to the users. For example, a free text space for journaling or positive quotes and reminders that are specific to that user would be welcome.

‘*Even if you got a quote like every day, it’s be nice to read something like that…*’ ‘*Quotes like that sometimes are just what you need when you’re in the bad space*.’

[W2, P1]

*That’s what I like about the Flo app because even though you have to have the premium feature to access it if it was different for this if you have the app you have the access to this because on Flo if there’s any changes it recommends stuff for you it recommends like meditations, it recommends articles and it says you’ve tracked acne like three times here’s some tips and it’s all from clinicians*.

[W2, P1]

Furthermore, the participants expressed interest in easy access to accredited resources on everything relating to the menstrual cycle, PMDD, and specific symptoms they are experiencing. By being instantly signposted to helpful websites or forums, they could come away from using the app with a mostly positive and educational experience, thus increasing the likelihood they would use it again.

*I love that they [Clue] have a whole host of like blog, you know articles to just give you a bit more insight*

[I4]

## Discussion

### Principal Findings

The purpose of this study was to identify the areas of improvement for an existing diagnostic survey and mood or menstrual tracking apps, and to gather the insights from users with lived experience of PMDD to prioritize a list of design requirements for a future prototype. The perspectives given by the attendees of the interviews and workshops provided valuable insights on how to best develop a mood and menstrual tracking app for users with PMDD. Both studies showed that the participants could see a benefit in using an app for tracking their symptoms. However, if they were to engage with it daily, it needed to be simple to use and have features that would benefit their mental well-being, rather than only recording symptom severity. We discuss these findings in greater detail below and provide 21 design requirements to support the design of this app. These requirements are summarized in Table 1.

Ease of use emerged vital for ensuring prolonged interaction with a tracker app. Typically, when designing any type of app, developers do prioritize a simple user-friendly interface [19] However, for users with PMDD, this is especially important for several reasons.

**Table 1.** List of design requirements for the development of a premenstrual dysphoric disorder (PMDD)–specific mood and menstrual tracking app.

Area	Design requirements
Theme 1: ease of use is a key consideration for users with PMDD	An easily accessible home page with icons and text depicting the different options for use of app (ie, diary entry, cycle summary, resources, and settings). An existing profile for users so they do not have to fill in their personal information every time. Reminders to complete the diary that users can set themselves. They can choose to opt in or opt out and can also pick which time these reminders come through. An option to customize colors and themes of the app to one’s own preferences in the settings.
Theme 2: avoiding a reductionist approach for a broad range of symptoms	The app asks users at the start what their typical cycle length or range is when setting up the profile, which can be changed at any point in the settings. The app also learns the cycle length from the data input by the user. The option to add in multiple entries from one day. Include a free text area where users can provide any additional context on their day. An option to identify a particular day or month as nonrepresentative of their normal cycle so that it does not affect their summary report. Symptom severity scale rather than a simple “yes or no” option. Users can add in any additional symptoms they may be feeling or wish to track going forward. Users can opt out of any symptoms that may not seem relevant to them after 2 months of use (due to a minimum of 2 symptomatic cycles being required for a PMDD diagnosis [2]), which can be changed in the settings and the user will receive reminders monthly to review these.
Theme 3: recognizing the importance of correct language	Users can opt out of menstrual cycle forecasting if preferred. Users can opt out of questions that may be triggering, such as ones relating to suicidality or self-harm. The app should avoid euphemisms and opt for clear and concise wording throughout. For each symptom ratings, users can click on it and be provided with a more detailed description of what the question is referring to in a more practical context.
Theme 4: integrating features for the users’ benefits	The app will generate several visualizations that users can access and share, depicting their mood symptoms against the menstrual cycle stages and a written summary of the trends seen. Users can opt in to see positive or motivational quotes and upload their own. An additional/optional section of the app can include videos of guided meditation and suggested mindfulness activities. Based on user inputs, the app should suggest accredited resources on how to manage their symptoms. There should be an area in the app with links to forums and support groups.

First, users with PMDD during their symptomatic phase are likely to feel burdened by symptoms such as low mood and increased stress. This was regularly mentioned by our participants and is also supported by the literature [2]. Moreover, when comparing PMDD to other mood disorders, such as depression or anxiety, research has highlighted irritability as one of the more prominent symptoms [27,28]. Therefore, it seems likely that there is an increased risk of discontinued use being attributed to issues with functionality, which is typically seen with mobile health anyway [29]. Some examples that our participants provided were other apps not automatically saving entries, and not receiving expected reminders, which can further discourage people from using and trusting an app. In order to reduce these concerns, the app needs to be simple to navigate with minimal distractions and allow for users to opt out of features that are non-beneficial for them to save effort.

Second, being able to encourage daily use for at least 2 months is required for the diagnosis of PMDD. As with most mobile health tools, the platform must therefore be interesting and appealing to users in order to entice them to spend time on it. [30]. Adding notifications to reminder users can be extremely useful for this to prompt users to fill out their daily entries. Although caution needs to be taken when introducing reminders, as previous literature has shown that excessive reminders have adverse effects and are considered annoying [31]. To resolve this, the easiest solution is for users to be able to tailor the timing and frequency of these reminders thus ensuring they are not too intrusive and are considered helpful [32].

In terms of the appearance of the app itself, previous studies have highlighted that a lack of color of visuals puts people off from using it [33]. Allowing users to personalize colors or add pictures has been suggested previously as a means to improve interactivity with the app [19]. Given the suggestions from the participants and newly emerging research looking at the relationship between neurodivergence (ADHD and autism) and PMDD [26], there is an additional requirement for gamification and positive feedback to encourage prolonged app use [34]. Clear icons and buttons to help users navigate the app, and being able to change the color to their own preference is a simple way to facilitate attraction to the aesthetics of the app.

This idea links closely with a need for feedback and features that benefit the user. Previous research has highlighted that feedback is an integral part of behavior change, thus is useful to consider for better retention rates [35]. For mood and menstrual tracking apps, the most common request from potential users is a visual summary of how their mood symptoms have changed against the stages of the menstrual cycle. Attendees of the interviews and workshops also requested the addition of helpful or positive resources, which seems like a good suggestion to improve motivation for interacting with the app and has additional clinical benefits when research-supported tools are integrated [36].

### Comparison to Prior Work

PMDD is quite unique as a mood disorder in the way the symptoms present and also due to the diagnostic requirement of daily tracking for 2 months. Nonetheless, there have been previous apps that have successfully promoted daily interaction for a prolonged period with a focus on health management or habit forming. The majority have also placed value on usability, engagement, and behavior change in a way that mirrors our findings [37]. The most notable of these successes are with monitoring food consumption for diabetes, using electrocardiogram sensors for cardiac diseases, and analyzing audio recordings of a user’s breathing for lung diseases [38].

The nature of menstrual tracking apps leans into the collection of highly personal data [39], which has led to a lot of criticism on problematic data collections and sharing practices [40]. There is a wide range of uses and the potential of misuse for these data, and a lack of transparency upheld by existing apps has led to accusations of menstrual surveillance [41] and monetizing female health [42]. These issues reinforce the need for greater consideration and clarification with users on how data are managed but also illustrate why the app is beneficial.

By integrating the suggestions from potential users, recognizing the success of predeceasing apps, and being wary of data management, it is possible to develop an app for the diagnosis and management of PMDD. This ideally will help educate researchers and medical professionals alike, while also having therapeutic benefits for the user.

### Limitations and Future Work

The main limitation of the study was the small sample size, which was mostly a result of strict time constraints of the project. In addition, as highlighted throughout this paper, PMDD has a notable impact on individuals’ everyday lives, and consequently, we had many participants who did not feel well enough to attend their allotted interview or workshops, which is why one of the workshops only had 1 attendee. However, the numbers are typical of this type of formative, user-centered study and are enough to highlight the biggest issues [43]. As detailed in the *Methods* section, the participants were recruited from the existing PREDDICT cohort. The participants for PREDDICT are recruited via social media and awareness events in collaborations with relevant charities. We did not take into consideration any educational or socioeconomic background, so there is a possibility that there could be a bias. The demographic questionnaire illustrated that all participants reported they were “White-British”; therefore, more effort to have a more diverse group could be taken for future studies. Nonetheless, there was a good range in ages and familiarity with technology, which allowed for a variety of perspectives. Future work should include people testing an existing prototype; this is something we are aiming to do next. This would allow potential users to interact with the app and give more practical suggestions on how it could be improved.

### Conclusion

People with PMDD use menstrual tracking apps to track, manage, and predict their mood and physical symptoms across the menstrual cycle. Only a few apps have all the necessary capabilities to fully capture the experience of someone with PMDD. Existing apps may fail to collect data, due to users not interacting with the app during their nonsymptomatic days. Also, they do not have PMDD-specific questions or ask for severity ratings. Based on the interviews and online workshops, we provided a list of 21 requirements for designing PMDD apps grounded in users’ experience and their needs. Having an app that is accessible, is visually interesting, and provides insights that could benefit the user’s mental well-being would encourage users to continuously input data, even when not feeling unwell. Moreover, by integrating existing PMDD symptoms measures such as the DRSP, users can more accurately record their symptom severity. This could have therapeutic benefits and help in conversations with medical professionals.

## Supplementary material

10.2196/59333 Multimedia Appendix 1 Participant information sheet (study 1).

10.2196/59333 Multimedia Appendix 2 Participants’ consent form.

10.2196/59333 Multimedia Appendix 3 Demographics questionnaire.

10.2196/59333 Multimedia Appendix 4 Study 1 interview guide.

10.2196/59333 Multimedia Appendix 5 Study 2 plan.

## Abbreviations

ADHD: attention-deficit/hyperactivity disorder
C-PASS: Carolina Premenstrual Assessment Scoring System
DRSP: Daily Record of Severity of Problems
*DSM-5*: *Diagnostic and Statistical Manual of Mental Disorders, Fifth Edition*
*ICD-11*: *International Classification of Diseases, Eleventh Revision*
PMDD: premenstrual dysphoric disorder
PMS: premenstrual syndrome
PREDDICT: premenstrual dysphoric disorder; indicators causes and triggers
