# Supplementary material for: Developing a Mood and Menstrual Tracking App for People With Premenstrual Dysphoric Disorder: User-Centered Design Study
Source: JMIR Form Res. 2024 Dec 24;8:e59333. doi: 10.2196/59333 (PMC11687174; doi:10.2196/59333)
Supplement: Multimedia Appendix 1 [file formative-v8-e59333-s001.docx]

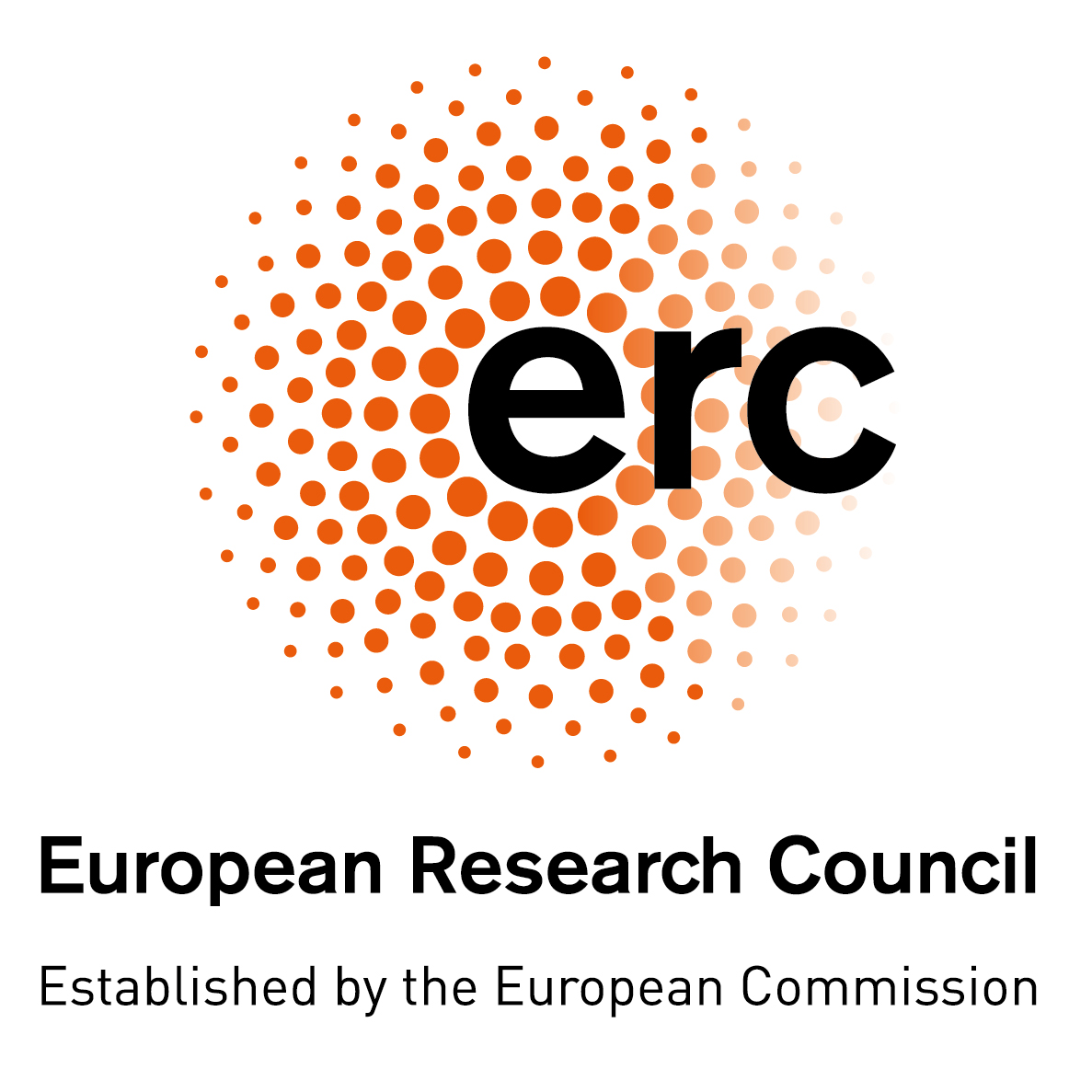

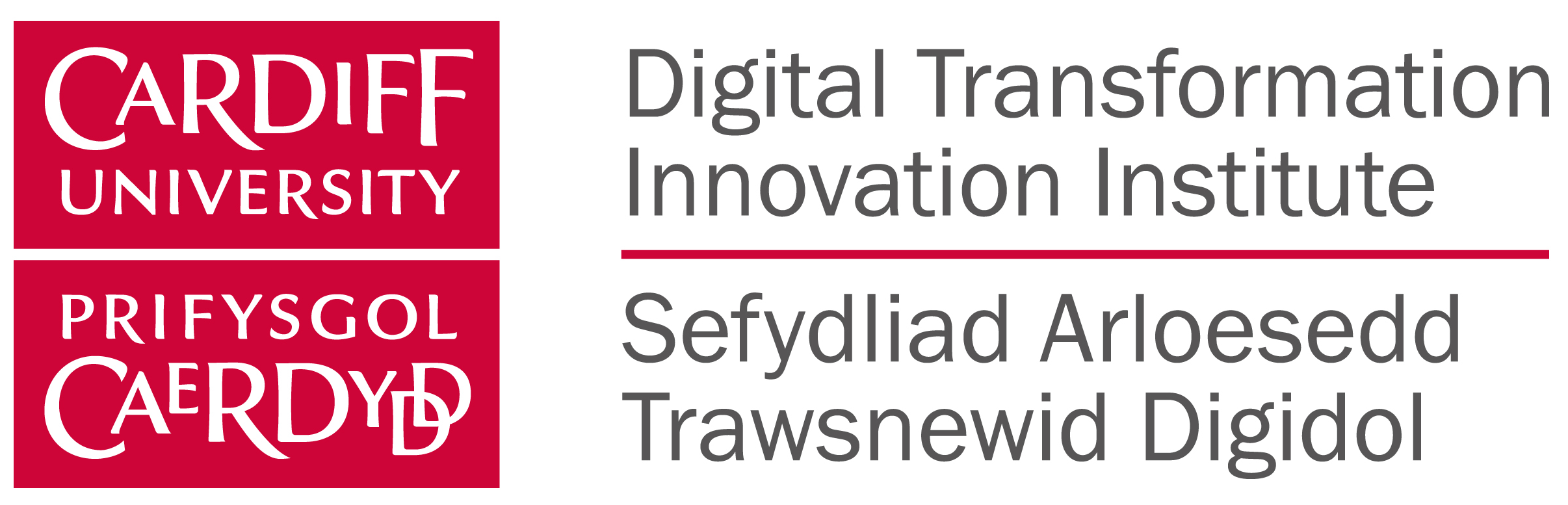


**Gathering requirements for an app that supports menstrual and mood tracking: Interview Study**

You are being invited to take part in a interview to discuss the development of a Mood Monitoring Application prototype. Before you decide whether or not to take part, you should understand why the interview is being held and what it will involve. Please take time to read the following information. Contact us if anything is not clear or if you would like more information. Take as much time as you need to decide whether or not you wish to take part.

1. **What is the purpose of the research?**

We are trying to develop a prototype for phone app for the monitoring mood symptoms and the menstrual cycle for our research into Premenstrual Dysphoric Disorder (PMDD) and/or Severe PMS.

Our aims are:

1. Understand issues with usability and engagement with the existing Mood Monitoring Diary that is used in our on-going study PreDDICT - *Premenstrual Dysphoric Disorder: Indicators, Causes and Triggers* research project.
2. Identify needs and desirable features that would support individuals in successfully completing 2 months of daily entries

Your participation in this study entirely optional and will not be compared to or impact your existing contributions to our research.

1. **Who is being asked to take part?**

You have been invited for one of two reasons

1. Because you have signed up to our PreDDICT - *Premenstrual Dysphoric Disorder: Indicators, Causes and Triggers* research project and completed a minimum of 2 entries in our existing ‘Mood Monitoring Diary’
2. Because you have lived experience of PMDD/Severe PMS and have completed a mood diary in the past

From your lived experience we hope to gain user insight and feedback to fulfil the aims outlined above, and co-create a successful app for the daily monitoring of PMDD symptoms and the menstrual cycle.

1. **Do I have to take part?**

No, your participation in this research project is entirely voluntary and it is up to you to decide whether or not to take part. If you decide to take part, we will discuss the research project with you and ask you to sign a consent form. If you decide not to take part, you do not have to explain your reasons and it will not affect your legal rights.

If you are a Cardiff University student, your involvement in this research project will have no effect on your education or progression through a degree course.

You are free to withdraw your consent to participate in the research project at any time, without giving a reason, even after signing the consent form or during the study.

1. **What does taking part involve?**

- The interview will take place online and will last approximately 30-45 minutes.
- You will be asked to provide feedback on the existing ‘PreDDICT Mood Monitoring Diary’ or other mood-menstrual tracking you have used, and share ideas on what you would like to be included.
- The interview topics will be emailed to you in advance so you can choose to prepare if you wish.

1. **Will I be paid for taking part?**

Yes, you will receive a £15 shopping voucher for your participation. You should understand that any data you give will be as a gift and you will not benefit financially in the future should this research project lead to the development of a new method/test/assessment.

1. **What are the possible benefits of taking part?**

Your help will also us in co-creating a tool that supports the existing PreDDICT project. The project is the largest study ever conducted on PMDD. GASSP aims to assess over 3,000 women with suspected psychiatric disorders associated with reproductive hormones and combine the assessment with DNA collection.

Additionally, we hope this will be the starting point for a grant application aiming to develop a NHS-friendly tools for the screening and diagnosis of reproductive mental disorders that is easy to use and facilitates ongoing engagement with the data collection necessary for the diagnosis.

1. **What are the possible disadvantages and risks of taking part?**

As outlined under the heading ‘Data Confidentiality’ below we can ensure that we will keep your information strictly confidential. You are more than welcome to put an alternative name when joining the call and support you if you need to leave the discussion at any point. If anything that comes up during the call is triggering or upsetting, we are happy to provide support and signpost you to any resources that may be beneficial to you.

1. **Data confidentiality**

All information collected from (or about) you during the research project will be kept confidential and any personal information you provide will be managed in accordance with data protection legislation. Please see ‘[What will happen to my Personal Data?](#personaldata)’ for further information. There are strict laws that safeguard your privacy at every stage. In accordance with GDPR and the Data Protection Act, your personal information will be kept confidential by assigning a unique study code to your sample and data. Your name or any identifying information will not be passed onto anyone. We will not pass on any of the information we collect unless we have serious concerns about your mental or physical health, or any concerns regarding the safety or wellbeing of others.

To make best use of resources we will share data with different groups of researchers from the NHS, universities and commercial companies, both within the UK and abroad. However, we would stress that those organizations will never obtain access to personal/ identifying information (for example, your name, address, date of birth).

1. **What will my data happen to my personal data?**

The discussion audio will be recorded and written up as a transcript. The transcript, alongside any written materials we will collect, will then be analysed to identify any common themes or ideas. From this we will develop a list of priorities that need to be considered by the app developers when creating the prototype.

Cardiff University is the Data Controller and is committed to respecting and protecting your personal data in accordance with your expectations and Data Protection legislation. Further information about Data Protection, including:

- your rights
- the legal basis under which Cardiff University processes your personal data for research
- Cardiff University’s Data Protection Policy
- how to contact the Cardiff University Data Protection Officer
- how to contact the Information Commissioner’s Office

may be found at <https://www.cardiff.ac.uk/public-information/policies-and-procedures/data-protection>

Within 2 weeks of data collection, the research team will anonymise all the personal data it has collected from, or about, you in connection with this research project, with the exception of your consent form and audio recordings. We will not include any names or identifiable information with the recordings. Your consent form and audio recordings will be retained for 5 years and may be accessed by members of the research team and, where necessary, by members of the University’s governance and audit teams or by regulatory authorities. Anonymised information will be kept for a minimum of 5 years but may be published in support of the research project and/or retained indefinitely, where it is likely to have continuing value for research purposes.

If you withdraw from the study at any time, all data recorded until that point will be immediately deleted. Please note that it will not be possible to withdraw any anonymised data that has already been published or in some cases, where identifiers are irreversibly removed during the course of a research project, from the point at which it has been anonymised.

1. **Declining and withdrawing from the study**

You do not have to take part in this study. If you do decide to take part you are still free to withdraw at any time without giving a reason. A decision to withdraw at any time, or a decision not to take part, will not alter the care you receive. If you decide to withdraw from this study, all details and information you have provided will be destroyed. These will not be used further in the research. In the unlikely event that you lose capacity, the research team will retain your identifiable sample and data and continue to use it in the research.

1. **What happens when the study is finished?**

This is a long-term study that will allow us to develop an prototype app for the monitoring of mood symptoms alongside tracking the menstrual cycle.

You will not receive specific results or feedback about your clinical information or sample. You will not have any claim to any future commercial use of results from the study in which your data has been used.

1. **What will happen to the results of the study?**

It is our intention to review the information we have collected and analyse the themes and ideas that come up during the discussion. These will be used to inform the app developer on what features are important to use when creating the app prototype. We may also publish the results of this thematic analyses in academic journals and present findings at conferences. Participants will not be identified in any report, publication or presentation.

We will keep in touch with you via our website and by sending an annual newsletter to let you know how the research is going and to ask you to let us know if your contact details change.

1. **Who is organising the research and why?**

This study is led by Katarzyna Stawarz (*School of Computer Science and Informatics)* and the partners in project are Prof Arianna Di Florio *(Department of Psychological Medicine and Clinical Neuroscience*) and Chloe Apsey *(Department of Psychological Medicine and Clinical Neuroscience).*The project is funded by the Digital Transformation Innovation Institute.

1. **Future research opportunities**

We may contact you again about other related research. You will be free to decline if you do not want to take part in these other research opportunities – just as you are free to withdraw from this study at any time.

1. **Who has reviewed this study?**

This research project has been reviewed and given a favourable opinion by the COMSC Research Ethics Committee, Cardiff University, project COMSC/Ethics/2023/076.

1. **What if there is a problem?**

If you wish to complain, or have grounds for concerns about any aspect of the manner in which you have been approached or treated during the course of this research, please contact Chloe Apsey or Dr Katarzyna Stawarz; please see section 17 for our contact details. If your complaint is not managed to your satisfaction, please contact the School Ethics Committee at [comsc-ethics@cardiff.ac.uk](mailto:comsc-ethics@cardiff.ac.uk).

If you are harmed by taking part in this research project, there are no special compensation arrangements. If you are harmed due to someone's negligence, you may have grounds for legal action, but you may have to pay for it.

1. **If you have further questions about the study please contact the study team:**

| **NCMH Team**  National Centre for Mental Health  Cardiff University  Hadyn Ellis Building,  Maindy Road, Cathays,  Cardiff CF24 4HQ  **Phone**029206 88922  **Email**   [reproductivementalhealth@cardiff.ac.uk](mailto:reproductivementalhealth@cardiff.ac.uk) | **COMSC Team**  School of Computer science  Abacws,  Senghennydd Road,  Cathays,  Cardiff, CF24 4AG  **Phone +44 (0)29 2251 0037**  **Email** [**stawarzk@cardiff.ac.uk**](mailto:stawarzk@cardiff.ac.uk) |
| --- | --- |

**If you would like to discuss this study with someone independent of the study please contact:**

**Vanessa Davies - Institute Manager**

Neuroscience and Mental Health Research Institute

3rd Floor, Hadyn Ellis Building

Maindy Road

CARDIFF

CF24 4HQ

**Phone**029 20688340

**Email** [daviesvj@cardiff.ac.uk](mailto:daviesvj@cardiff.ac.uk)
