# Supplementary material for: Developing a Mood and Menstrual Tracking App for People With Premenstrual Dysphoric Disorder: User-Centered Design Study
Source: JMIR Form Res. 2024 Dec 24;8:e59333. doi: 10.2196/59333 (PMC11687174; doi:10.2196/59333)
Supplement: Multimedia Appendix 2 [file formative-v8-e59333-s002.docx]

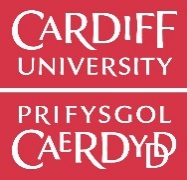


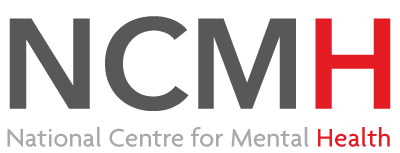


***Title of research project: Gathering requirements for an app that supports menstrual and mood tracking – Interview Study***

*SREC reference and committee: School of Computer Science and Informatics Research Ethics Committee, project COMSC/Ethics/2023/076*

*Name of Chief/Principal Investigator: Dr Katarzyna Stawarz & Prof Arianna Di Florio*

|  | **Please initial each box as appropriate** |
| --- | --- |
| 1. I have read the attached information sheet (version 1.0, 03/05/2023) and have had the opportunity to ask questions about the project. I know how to contact the research team if I need to and have a copy of the information sheet |  |
| 1. I consent to the processing of my personal information (age, gender, ethnic background, diagnosis) for the purposes explained to me.  I understand that such information will be held in accordance with all applicable data protection legislation and in strict confidence, unless disclosure is required by law or professional obligation. |  |
| 1. I understand that the discussion audio may be recorded and written up as a transcript. However, names and personal information will not be stored with this. |  |
| 1. I understand that participation in this project is voluntary and that I am free to withdraw from the study without giving a reason and without any medical treatment or care I may receive being affected. |  |
| 1. I understand that I will not benefit financially should this research lead to the development of a new treatment or medical test. By signing this form I consent to, and make no claim of right in, the unrestricted use of such data and samples for genetic and other research |  |
| 1. I agree for analysis of my input into the discussion to be carried out by researchers other than the core NCMH research team. This includes individuals working abroad, including researchers working for commercial companies. |  |
| 1. I understand that I will not receive specific results or feedback from my participation in the NCMH study. |  |
| 1. I agree to be contacted about this and related studies and understand that I am free to decline if I do not wish to participate in these future research opportunities. |  |
| 1. I understand my participation in this study will not impact my contributions to the existing PreDDICT study, and my ideas and discussion points brought up in the session will not be compared to or link back to my participation in the PreDDICT project. |  |
| 1. I agree to take part in this study. |  |

*Name of participant (print) Date Signature*

*Name of person taking consent Date Signature*

*(print)*

***_________________________***

*Role of person taking consent*

*(print)*

***THANK YOU FOR PARTICIPATING IN OUR RESEARCH***

***YOU WILL BE GIVEN A COPY OF THIS CONSENT FORM TO KEEP***
