# Supplementary material for: Developing a Mood and Menstrual Tracking App for People With Premenstrual Dysphoric Disorder: User-Centered Design Study
Source: JMIR Form Res. 2024 Dec 24;8:e59333. doi: 10.2196/59333 (PMC11687174; doi:10.2196/59333)
Supplement: Multimedia Appendix 3 [file formative-v8-e59333-s003.docx]

*This questionnaire will be set up as a Microsoft Form and the link will be emailed to participants prior to each study.*

1. Name:

[___free text__]

1. Age

- 18-25
- 26-33
- 34-41
- 42-50
- Over 50
- Prefer not to answer

1. What is your gender identity? **(Please select one, however if multiple fit your identity, please select other and list them)**

- Male
- Female
- Transgender Male
- Transgender Female
- Gender variant/Non-conforming
- Other [___*free text*__]
- Prefer not to answer

1. What is your ethnic origin? **(Please select one)**

| White | - English - Welsh - Scottish - Irish - Northern Irish - Gypsy or Irish Traveller - Any other white background |
| --- | --- |
| Asian | - Indian - Pakistani - Bangladeshi - Chinese - Any other Asian background |
| Black/Black British | - African - Caribbean - Any other Black/African/Caribbean background |
| Mixed/Multiple Ethnic groups | - White and Black Caribbean - White and Black African - White and Asian - Any other mixed of multiple ethnic backgrounds |
| Middle Eastern Ethnic Group | - Arab - Other middle eastern group |
| Other Ethnic group | - Other ethnic group |
| Unknown | - Unknown - Prefer not to say |

Which of the following statements apply to you? **(Please Select one)**

- - I am currently receiving or have previously received a diagnosis and/or treatment for PMDD/severe PMS by a healthcare professional
  - I have been diagnosed with PMDD/severe PMS by a healthcare professional
  - I am currently undergoing investigations for PMDD/severe PMS
  - I think I have PMDD/severe PMS, but never sought professional help
  - None of the above

Have you completed a “mood diary”/daily tracking of symptoms in the past? **(Please select one)**

- - - - Yes
      - No
      - Not Sure
