# Supplementary material for: Developing a Mood and Menstrual Tracking App for People With Premenstrual Dysphoric Disorder: User-Centered Design Study
Source: JMIR Form Res. 2024 Dec 24;8:e59333. doi: 10.2196/59333 (PMC11687174; doi:10.2196/59333)
Supplement: Multimedia Appendix 4 [file formative-v8-e59333-s004.docx]

**Interview guide**

*Thank you for agreeing to provide feedback on your experiences with RedCAP. I would like to ask you a few questions about your experience. This interview should take about 30-45 minutes.*

***Experience with RedCAP***

1. Can you tell us about your overall experience with RedCAP? What worked well, what didn’t?
2. How long were you using it?
   1. If dropped out – Why did you stop using it?
3. Is there anything we could change in RedCAP to make it more useful?

***Questions about specific features***

*Now I have a few questions about specific RedCAP functionality.*

1. What did you think about the reminder functionality? Was it useful / How could it be improved?
2. What did you think about the questionnaire?
3. We know that other users found RedCAP difficult to use. Did you experience any difficulties?
4. Was there anything you liked about RedCAP?

***Potential improvements***

*We are hoping to design an app that supports data collection to support the diagnosis and also provides additional features that may be useful for people with PMDD. I would like to talk to you about menstrual and mental health apps in general.*

1. Have you ever used any apps for support mental or menstrual health? E.g. trackers?
   1. If yes – which ones and why?
   2. If not – why not?
2. Was there any functionality you found in particular useful?
3. Was there anything that you needed but perhaps wasn’t available in any of these apps?

***SUS questionnaire***

*Now let’s get back to RedCAP. I’m going to read 10 statements. Please think about the RedCAP survey and tell me whether you strongly agree or agree, strongly disagree or disagree, or neither agree or disagree. It would be useful if you could explain your answers.* [researcher to prompt explanations for each answer]

1. I think that I would like to use the RedCAP survey frequently.

Strongly disagree 1 2 3 4 5 Strongly agree

2. I found the RedCAP survey unnecessarily complex.

Strongly disagree 1 2 3 4 5 Strongly agree

3. I thought the RedCAP survey was easy to use.

Strongly disagree 1 2 3 4 5 Strongly agree

4. I think that I would need the support of a technical person to be able to use the RedCAP survey.

Strongly disagree 1 2 3 4 5 Strongly agree

5. I found the various parts of the RedCAP survey were well integrated.

Strongly disagree 1 2 3 4 5 Strongly agree

6. I thought there was too much inconsistency.

Strongly disagree 1 2 3 4 5 Strongly agree

7. I imagine that most people would learn to use RedCAP survey very quickly.

Strongly disagree 1 2 3 4 5 Strongly agree

8. I found the RedCAP survey very awkward to use.

Strongly disagree 1 2 3 4 5 Strongly agree

9. I felt very confident using the RedCAP survey.

Strongly disagree 1 2 3 4 5 Strongly agree

10. I needed to learn a lot of things before I could get going with the RedCAP survey.

Strongly disagree 1 2 3 4 5 Strongly agree

***Finishing questions***

- In general, what do you think of using technology to support mental and menstrual health?
- Is there anything else you would like to add that hasn’t been covered?
