# Supplementary material for: Developing a Mood and Menstrual Tracking App for People With Premenstrual Dysphoric Disorder: User-Centered Design Study
Source: JMIR Form Res. 2024 Dec 24;8:e59333. doi: 10.2196/59333 (PMC11687174; doi:10.2196/59333)
Supplement: Multimedia Appendix 5 [file formative-v8-e59333-s005.docx]

**Co-design workshop guide**

*The workshop will take place online and will last 2 hours with a 10 minute break in the middle. We will be using Zoom/Teams and a Miro board to support group activities.*

***Introductions [15 min]***

1. Welcome and introductions
2. Short overview of the project
3. Overview of the session and participant Q&A
4. Collection of any missing consent forms
5. Recording starts

***Activity 1 [15 min]***

1. Introduction to the 1^st^ activity
2. Introduction to the Miro board
3. Task: participants make a list (on Miro) of all tools (technology and otherwise) they used for tracking their menstrual and mental health
4. Short discussion about the results

***Activity 2 [15 min]***

1. For each identified app, participants make a list of:
   1. Things liked about them
   2. Things they didn’t like
   3. Things they thought were missing or would make the app more useful/relevant
2. Short discussion about the result

*[10 min break]*

***Activity 3 [30 min]***

1. Participants are put into groups (breakout rooms)
2. Groups work together to design an ideal app
   1. Based on the list from Activity 2, they identify top 5 features the app should have
   2. They discuss and decide how the app could work
3. Facilitators visit the rooms to provide prompts and suggestions

***Final discussion [20 min]***

1. Group report back and present their ideas
2. General discussion about:
   1. Common points between the groups and key features
   2. What would make menstrual health apps more useful for participants
   3. Any challenges and issues that should be covered by such apps

***Wrap up [5 min]***

1. Final remarks
2. Stop recording
